# HEALTH PROMOTION AS CATALYST: CHALLENGES AND COMPLEXITIES OF HEALTHCARE AMONG OLDER ASIAN IMMIGRANTS

**DOI:** 10.1093/geroni/igad104.3381

**Published:** 2023-12-21

**Authors:** Sabita Shrestha, Jae Kim, Mary Hogue

**Affiliations:** Western Kentucky University, Bowling Green, Kentucky, United States; Western Kentucky University, Bowling Green, Kentucky, United States; Primary Health Clinic, Las Vegas, Nevada, United States

## Abstract

Older adults are the most disadvantaged, vulnerable, and marginalized groups in the world. They often experience various challenges and complexities in navigating healthcare and other social care. Needless to say, these complexities are profound, especially, among older Asian immigrants in navigating healthcare and social care compared to other populations in the US. They often face various sociocultural challenges including structural barriers in search of proper healthcare and accessibility to care. The studies have shown older Asian immigrants are reluctant to seek or access needed care due to structural barriers poised against them. In addition, lack of cultural awareness and understanding by healthcare professionals further impedes accessing timely healthcare. Despite the complexities of healthcare navigation, health education and promotion play an important role as catalysts in disseminating health information relevant to healthcare and structural barriers to older Asian immigrants. Based on ecological framework, this presentation will showcase a holistic approach to health education intervention and promotion aimed to minimize complexities of healthcare navigation and structural barriers towards older Asian immigrants in the US.

